# Genes implicated in stem cell identity and temporal programme are directly targeted by Notch in neuroblast tumours

**DOI:** 10.1242/dev.126326

**Published:** 2016-01-15

**Authors:** Evanthia Zacharioudaki, Benjamin E. Housden, George Garinis, Robert Stojnic, Christos Delidakis, Sarah J. Bray

**Affiliations:** 1 Department of Physiology, Development and Neuroscience, University of Cambridge, Cambridge CB2 3DY, UK; 2 Institute of Molecular Biology and Biotechnology, FORTH-Hellas, Heraklion, Crete 70013, Greece; 3 Department of Biology, University of Crete, Heraklion, Greece GR71409; 4 Cambridge Systems Biology Centre, University of Cambridge, Cambridge CB2 1QR, UK

**Keywords:** *Drosophila*, Neuroblast, Notch, Gene regulation, Stem cell

## Abstract

Notch signalling is involved in a multitude of developmental decisions and its aberrant activation is linked to many diseases, including cancers. One example is the neural stem cell tumours that arise from constitutive Notch activity in *Drosophila* neuroblasts. To investigate how hyperactivation of Notch in larval neuroblasts leads to tumours, we combined results from profiling the upregulated mRNAs and mapping the regions bound by the core Notch pathway transcription factor Su(H). This identified 246 putative direct Notch targets. These genes were highly enriched for transcription factors and overlapped significantly with a previously identified regulatory programme dependent on the proneural transcription factor Asense. Included were genes associated with the neuroblast maintenance and self-renewal programme that we validated as Notch regulated *in vivo*. Another group were the so-called temporal transcription factors, which have been implicated in neuroblast maturation. Normally expressed in specific time windows, several temporal transcription factors were ectopically expressed in the stem cell tumours, suggesting that Notch had reprogrammed their normal temporal regulation. Indeed, the Notch-induced hyperplasia was reduced by mutations affecting two of the temporal factors, which, conversely, were sufficient to induce mild hyperplasia on their own. Altogether, the results suggest that Notch induces neuroblast tumours by directly promoting the expression of genes that contribute to stem cell identity and by reprogramming the expression of factors that could regulate maturity.

## Abstract (precis)

**Summary:** Genome-wide screens identify direct targets of Notch involved in promoting neural stem cell tumours in *Drosophila,* tying Notch into the transcription factor network coordinating these stem cells.

## INTRODUCTION

The Notch pathway is a cell communication mechanism that is involved in many developmental decisions and in stem cell homeostasis of adult tissues. Furthermore, abnormal Notch activity is linked to various diseases, including several forms of cancer. Indeed, in some cancers Notch is thought to have a role in the initiation and maintenance of cancer stem cells (CSCs) (Capaccione and Pine, 2013; Ntziachristos et al., 2014). One context in which high Notch activity causes stem cell hyperplasia, with similarities to CSCs, is in *Drosophila* neural stem cells, the so-called neuroblasts (NBs). Notch is normally active in NBs but is rapidly inactivated in their progeny. Sustained activity of the pathway in the NB lineages results in brain tumours, where the overproliferation of NBs at the expense of neurons gives rise to large NB masses in the brain that compromise the survival of the animals to adulthood (Bowman et al., 2008; Wang et al., 2006; Weng et al., 2010). It is therefore important to understand how sustained Notch activity alters the balance between self-renewal and differentiation to result in tissue tumorigenesis.

In normal circumstances, the larval NBs undergo repeated rounds of asymmetric division to generate neurons appropriate for the adult CNS. At each division the larger cell maintains NB properties and regrows to sustain many rounds of division (Knoblich, 2008; Sousa-Nunes and Somers, 2013). The majority are Type I NBs, identified by expression of the transcription factors (TFs) Deadpan (Dpn) and Asense (Ase), whose small daughter cell, the ganglion mother cell (GMC), divides terminally to produce two neurons and/or glia. A small number of NBs, the so-called Type II NBs (eight per brain lobe), express Dpn but not Ase and follow a more complex pattern of division. When these divide asymmetrically, their smaller daughter is an immature intermediate neural progenitor (INP), which reaches maturation within a few hours and then itself divides asymmetrically a few times. In this case, the daughter is a GMC similar to that of Type I NBs. The existence of INPs enables Type II NBs to generate a large number of progeny in a short period of time (Bayraktar and Doe, 2013; Bello et al., 2008; Boone and Doe, 2008; Bowman et al., 2008; Izergina et al., 2009; Kang and Reichert, 2014; Knoblich, 2008). At the end of larval life, both Type I and Type II NBs exit the cell cycle and cease proliferation, under the influence of temporal factors (Chai et al., 2013; Maurange et al., 2008), the steroid hormone ecdysone (Homem et al., 2014) and other cues (Chai et al., 2013).

Notch pathway activity is detected in NBs and contributes to their maintenance. During mitosis, one of the key determinants that is segregated asymmetrically into the GMC daughter is Numb, a potent inhibitor of Notch signalling (Babaoglan et al., 2009; Connor-giles et al., 2003; Guo et al., 1996; Le Borgne et al., 2005; Rhyu et al., 1994; Spana and Doe, 1996; Wang et al., 2006). Perturbations in Numb function lead to uncontrolled proliferation of NBs and the formation of brain tumours. This is largely caused by the ectopic Notch activity that ensues, a condition that is mimicked by expression of a constitutively active Notch fragment (Bowman et al., 2008; Wang et al., 2006; Weng et al., 2010). Upon interaction with its ligands [Delta (Dl) or Serrate (Ser)], the Notch receptor undergoes two proteolytic cleavages to release the Notch intracellular domain (Nicd), which translocates into the nucleus where it interacts with the CSL (also known as RBPJ) DNA-binding protein {Suppressor of Hairless [Su(H)] in *Drosophila*} and activates the transcription of target genes (Bray, 2006; Kopan and Ilagan, 2009). Expression of Nicd or of a transmembrane fragment mimicking the first ligand-activated cleavage (NΔecd), results in similar brain tumours to those caused by loss of Numb.

One significant target of Notch activity in NBs is *Enhancer of spilt mγ* [*E(spl)mγ-HLH*], a HES family gene that is dependent on Notch for expression (Almeida and Bray, 2005). However, mutations removing the entire *E(spl)* complex [*E(spl)-C*] of Notch-responsive genes have only minor effects on NB maintenance, suggesting that additional targets exist. Indeed, *E(spl)mγ-HLH* appears to function semi-redundantly with *dpn*, another HES family gene, which has both Notch-dependent and Notch-independent modes of regulation in NBs (San-Juán and Baonza, 2011; Zacharioudaki et al., 2012; Zhu et al., 2012). In addition, expression of the zinc-finger protein Klumpfuss (Klu) may also be Notch regulated in this context (Berger et al., 2012; Xiao et al., 2012). Overexpression of *E(spl)mγ-HLH*, *dpn* or *klu* can cause NB hyperplasia (Berger et al., 2012; San-Juán and Baonza, 2011; Xiao et al., 2012; Zacharioudaki et al., 2012); however, their effects are generally weaker or more spatially limited than that of Nicd or NΔecd. It therefore appears that these Notch targets do not account for the full scope of Notch functions in normal NBs, nor in the hyperactive Notch-induced NB tumours.

To characterise the repertoire of genes activated by Notch in overproliferating NB tumours we compared the transcriptional profiles from the CNS of Notch-induced NB hyperplasia with wild type (WT) and integrated these data with maps of the regions bound by Su(H) in the Notch hyperplasia. The Notch targets identified in this way were highly enriched in genes encoding TFs associated with NB maintenance and the self-renewal programme, as well as TFs that are implicated in the temporal programming of the stem cells. Validating these targets and their functions *in vivo* suggests that stemness and temporal TFs might cooperate to sustain Notch-induced hyperplasias. Furthermore, the redundancy between the identified targets potentially gives a robustness to the signalling output that could explain why the previously known targets are insufficient to account for the Notch activation phenotype.

## RESULTS

### Identification of Notch target genes involved in NB hyperplasia

**Fig. 1.**
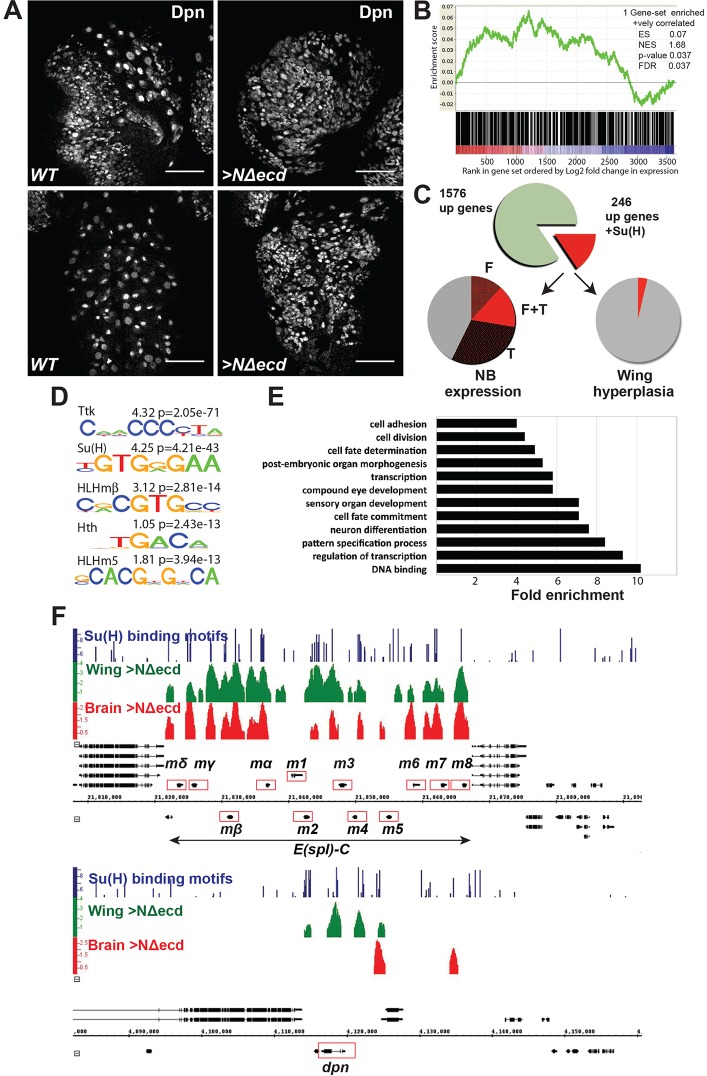
**Identification of target genes in Notch-induced NB hyperplasia.** (A) Wild-type (WT) and hyperplastic (NΔecd; expressed using *grhNB-Gal4*) *Drosophila* CNS. Dpn marks NBs in brain lobes (top) and VNC (bottom). Scale bars: 50 µm. (B) GSEA plot of enrichment scores (classic scoring approach) for Su(H)-bound genes [defined as genes within 20 kb of an Su(H) peak] relative to genes showing expression changes, pre-ranked by log_2_ fold change (FC) (3717 genes, FDR≤0.1) indicates that the Su(H) gene set is significantly enriched in the upregulated genes. ES, enrichment score, which indicates the degree of overrepresentation at the top of the ranked list; NES, normalised enrichment score; *P*-value, nominal *P*-value; FDR, false discovery rate. (C) The proportion of upregulated genes (1576 genes, FDR≤0.1) with Su(H) binding (red, 246 genes) and their relationship to genes with known expression in NBs and to Notch targets in wing hyperplasia. NB expression was determined by RNAseq of FACS sorted cells [F (Berger et al., 2012)] or by targeted DamID with PolII [T (Southall et al., 2013)], or both (F+T). (D) Sequence logo of motifs enriched in Su(H)-bound fragments. Enrichment for the Su(H) motif is more significant than in related ChIP datasets (e.g. Skalska et al., 2015). (E) Functional characteristics of 246 putative NB Notch target genes. GO categories are ranked by enrichment (≥2-fold enrichment, *P*≤0.05; overlapping processes were grouped and the most enriched selected). (F) Examples of Notch-regulated genes in genomic regions spanning *E(spl)-C* (top) and *dpn* (bottom). Graphs depict Su(H)-bound regions (enrichment relative to input AvgM, scale log_2_ 0-4) in NΔecd brains (red) and in NΔecd wing discs (green). Blue bars, conserved Su(H) binding motifs identified using Patser; bar height represents the Patser score, scale 5-9.79 (Hertz and Stormo, 1999). Gene models are depicted in black.

Constitutively active Notch (NΔecd) results in NB overproliferation at the expense of neurons (Bowman et al., 2008; Wang et al., 2006). To identify genes acting downstream of Notch to produce NB hyperplasia, we first characterised the population of RNAs showing elevated expression, since the Nicd complex results in transcriptional activation (Fig. 1A). NΔecd was expressed for 24 h in larval NBs (via *grhNB-Gal4 Gal80ts*) to produce CNS dominated by Dpn-expressing NBs (Fig. 1A), which included many Ase^−^ Type II NB-like cells as well as Ase^+^ Type I NB-like cells (Fig. S1A). A comparison of their RNA expression profile with that of control CNS of a similar stage identified 1576 upregulated transcripts in NΔecd hyperplastic CNS (FDR≤0.1) (Table S1). We note that, as this reflects changes in the entire CNS transcriptome, the effects of NΔecd will be attenuated by the non-NB cells and there will be indirect effects from the altered ratios of cell types in the hyperplastic brains.

To distinguish which genes may be directly regulated by Notch activity, genomic regions occupied by Su(H) in hyperplastic brains were identified by ChIP. The 595 bound regions were significantly enriched for sequences matching the consensus Su(H) binding motif YGTGRGAA (*P*=4.11×10^−43^), strengthening the likelihood that they represent bona fide binding sites. Other enriched motifs included those for Tramtrack (Ttk), which is expressed in glial cells (Badenhorst, 2001) and may thus repress the enhancers in those lineages, for bHLH repressor proteins (CRCGTG) and for the homeodomain protein Homothorax (Hth) (TGACA), which is discussed further below (Fig. 1D, Fig. S1C).

Our criteria for direct Notch-regulated genes are that they should be both upregulated in the Notch-induced hyperplasia and associated with Su(H)-occupied regions. This implies that Su(H)-bound genes should be enriched among those that are upregulated by NΔecd, which we confirmed by two approaches. First, gene set enrichment analysis (GSEA) (Mootha et al., 2003; Subramanian et al., 2005) using a pre-ranked gene list from the expression analysis (3717 genes, FDR≤0.1) indicated a significant enrichment of Su(H)-bound genes among those with upregulated mRNAs (normalised enrichment score=1.68, *P*=0.037; Fig. 1B). Second, a comparison of the Su(H)-bound genes with gene lists generated from 10,000 randomly generated ChIP peak sets demonstrated that the former were preferentially enriched among the more highly upregulated genes [log_2_ fold change (FC)>0.5; see Materials and Methods and Table S3].

By intersecting the 1576 upregulated genes with those located within 20 kb of Su(H) peaks, we identified a set of 246 candidates for direct Notch targets in the CNS stem cells, of which 185 had log_2_ FC>0.5 (Fig. 1C, Table S2). These included *E(spl)mγ-HLH* and *dpn* (Fig. 1F), two genes previously associated with Notch regulation in larval NBs. Furthermore, the gene set was significantly enriched in NB-expressed genes (*P*=5.47×10^−9^), based on two different experiments in which NB transcription profiles were analysed (Fig. 1C) (Berger et al., 2012; Southall et al., 2013). Conversely, the gene set was not enriched for genes expressed in sensory organ proneural clusters (Reeves and Posakony, 2005), where Notch has an opposing function and is off in the neural precursor.

Functional characteristics of the 246 putative direct Notch targets were assessed using gene ontology (GO) and protein domain annotations (Fig. 1E). Besides general development-related categories (‘post-embryonic organ morphogenesis’, ‘cell fate determination’), enriched categories included ‘regulation of transcription’ and ‘DNA binding’, along with three neurogenesis-related categories (‘neuron differentiation’, ‘sensory organ development’, ‘compound eye development’; Fig. 1E). Several targets in the transcription category have been implicated in NB maintenance [*E(spl)mγ-HLH*, *dpn*, *klu*, *wor*, *grh* (Almeida and Bray, 2005; Berger et al., 2012; Cenci and Gould, 2005; San-Juán and Baonza, 2011; Song and Lu, 2011; Xiao et al., 2012; Zacharioudaki et al., 2012; Zhu et al., 2012)] or in their temporal regulation [*cas*, *svp*, *hth* (Li et al., 2013; Maurange et al., 2008)].

Strikingly, Notch-regulated genes in NB hyperplasia were generally different from those in an epithelial hyperplasia caused by excessive Notch activity in wing imaginal discs (Djiane et al., 2013). First, the overall functional characteristics differed: in NB hyperplasia the target genes were associated with cell fate commitment and transcription (Fig. 1E), whereas in epithelial hyperplasia they were enriched for signalling pathways and proliferation control (Djiane et al., 2013). Second, only nine genes appeared to be directly Notch induced in both conditions (Fig. 1C; *P*=0.028). Even when genes from a similar Su(H)-induced wing disc hyperplasia were added, the overlap only increased to 18 genes, although this is a significant enrichment demonstrating that the responses are related (*P*=1.091×10^−6^). Besides *E(spl)mγ-HLH*, the overlap included *dpn* (also HES related) and *Myc*. The latter is widely regulated by Notch in many contexts both in flies and mammals (Djiane et al., 2013; Klinakis et al., 2006; Krejcí et al., 2009; Weng et al., 2006). Finally, even for those genes that were regulated in both tissues, the Su(H) binding profiles differed (e.g. Fig. 1F). For example, in CNS, Su(H) was detected at a region 5′ of the *dpn* gene body, which overlapped with a previously identified NB enhancer (San-Juán and Baonza, 2011), whereas in wing-discs it was predominantly bound at an intronic enhancer (Babaoğlan et al., 2013). These results demonstrate that Notch responses can differ extensively, even in circumstances when the eventual outcomes for the tissue are similar (i.e. tissue overgrowth).

### Notch regulates genes linked to stem cell identity

**Fig. 2.**
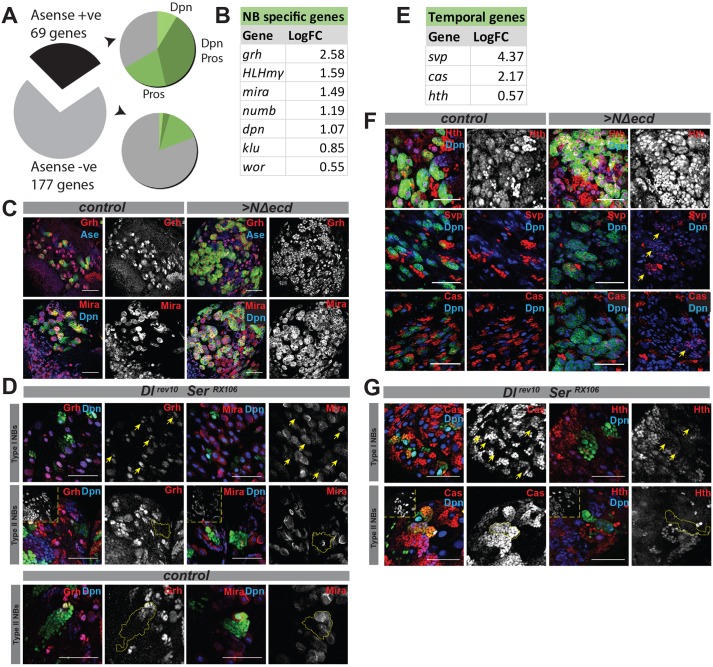
**Notch regulates the NB stem cell network as well as temporal genes.** (A) A significant proportion of Notch NB targets overlap with Ase-regulated genes (black; *P*=4.747×10^−16^). This subset is enriched in Dpn (light green), Dpn and Pros (dark green), and Pros (mid-green) regulated genes (*P*=7.794×10^−26^) compared with the Ase^−^ subset (*P*=0.005). Both subsets show similar enrichments for NB-expressed genes (data not shown). (B) Examples of NB-specific genes upregulated in Notch-induced hyperplasia and associated with Su(H) peaks; log_2_ FC relative to controls. All are in the Ase^+^ subset of NB targets. (C) Grh and Mira are upregulated by ectopic Notch activity. Expression of Grh (top row; red, white) and Mira (bottom row; red, white) in WT (control) and NΔecd-expressing NBs, marked by CD8-GFP (green). In this and subsequent figures Dpn or Ase marks NBs as indicated (Ase also labels GMCs). (D) Sensitivity of Grh and Mira expression to disruption of the Notch pathway. Grh (left panels; red, white) or Mira (right panels; red, white) expression in GFP-marked clones mutant for *Dl^rev10^ Ser^RX106^* (top two rows; green) or WT (bottom row; green). Yellow arrows indicate NBs from mutant Type I lineages; yellow outlines mark mutant or control Type II lineages. Insets are Dpn only, to show INP numbers in mutant lineages. Type II lineages were scored as having altered expression if levels in NBs or INPs were reduced and/or if fewer Dpn^+^ INPs expressed the gene of interest. (E) Temporal genes included among the Notch targets; log_2_ FC relative to controls. (F) Sporadic upregulation of Hth, Svp and Cas (red or white as indicated) in NBs expressing NΔecd (right panels; marked by CD8-GFP, green) compared with controls (left panels; CD8-GFP, green). Only Hth is detectable in some control NBs, where levels are low. With NΔecd expression, Hth is present at high levels in all supernumerary NB-like cells; Svp and Cas are expressed *de novo* in ectopic NB-like cells. (G) Disruption of Notch activity (*Dl^rev10^ Ser^RX106^*) does not perturb Hth or Cas expression in Type I (yellow arrows) or Type II lineages (yellow outline). GFP-marked clones (green) are stained for Cas or Hth (red or white). Insets are Dpn only, to show INP numbers in mutant lineages. Scale bars: 50 μm.

Two previous studies have investigated gene networks involved in regulating NBs. One evaluated the enrichment of RNAs in NBs compared with neurons in the larval CNS and then proposed a regulatory network of TFs (Berger et al., 2012). This TF network is significantly represented amongst our CNS direct Notch targets (*P*=7.278×10^−17^; Fig. S1B). Indeed, our results confirm Notch regulation of a core network of these genes. The second used the binding profile of the proneural protein Ase to identify genes involved in NB programming in the embryo (Southall and Brand, 2009). Again, the Notch target genes were highly enriched in these Ase-bound genes: 69/246 were identified targets of Ase (*P*=4.75×10^−16^; Fig. 2A). This subset of Notch targets was also enriched in genes bound by two other TFs involved in regulating NB lineages, namely Dpn and Pros (*P*=7.794×10^−26^; Fig. 2A) (Southall and Brand, 2009). By contrast, the 177 genes that were not Ase targets showed less overlap with Dpn/Pros regulation (*P*=0.005; Fig. 2A). Nevertheless, both Ase^+^ and Ase^−^ subsets were similarly enriched in NB-expressed genes, suggesting that the latter are also important in NBs.

Altogether, these comparisons reveal that a significant proportion of the Notch targets have been implicated in regulatory networks coordinating the NB stem cell programme (e.g. Fig. 2B). We therefore sought to verify their response to Notch signalling by testing whether expression from a subset (*grh*, *wor*, *mira*, *numb*) was modified when NBs were subjected to a pulse of NΔecd for 24 h. All four genes were expressed in overproliferating NB-like cells generated under these conditions (*grh*, *mira*, Fig. 2C; *wor*, *numb*, Fig. S2A). Indeed, Mira levels were significantly higher in the NΔecd lineages (Fig. S2C,D), although we note that, in general, the major effect is one of more cells expressing these genes rather than a large increase in expression levels per cell. Nevertheless, there is widespread expression of the stem cell genes in response to ectopic Notch activity *in vivo*, in agreement with the expression array results.

To test whether stem cell network genes are sensitive to reduced Notch function, we generated marked clones of cells mutant for *Dl*, *Ser* [which compromises Notch activity in NBs (Zacharioudaki et al., 2012)] or for *mastermind* (*mam*) (to prevent the transcriptional activation of targets). Such manipulations led to significantly altered expression of *mira* (100% of lineages, *n*=8; Fig. 2D), *grh* (85.72% of lineages, *n*=7; Fig. 2D), *wor* (60% of lineages, *n*=5) and *numb* (100% of lineages, *n*=7) in Type II lineages (Fig. S2B, Table S4). The effects in Type I lineages were more subtle, as *mira* was the only gene that was significantly reduced in expression (16.2% of lineages, *n*=99; Fig. 2D, Table S4), although the levels of Numb and Wor were also reduced in some clones. Altogether, these data are consistent with these genes being under Notch regulation in some NB lineages. The fact that their expression is not abolished upon Notch loss of function in Type I NBs might be the consequence of additional, compensatory regulatory inputs, as has been shown for *dpn* (San-Juán and Baonza, 2011; Zacharioudaki et al., 2012), or of Type I NBs being more resilient to reductions in Notch activity (Bowman et al., 2008; Wang et al., 2006).

### Notch regulates the expression of TFs implicated in temporal programming

In addition to genes implicated in NB identity, which are expressed consistently and specifically in NBs, Notch targets also included genes with more dynamic NB expression (Fig. 2E). Several of these are involved in temporal programming of NBs and their progeny (Maurange et al., 2008). In particular, *svp* and *cas* are expressed in NBs at mid-larval stages and regulate a change in the size and identity of the neurons produced, as well as determining the time that NBs will undergo cell cycle exit or Reaper/Hid/Grim-dependent apoptosis (Maurange et al., 2008). *hth* has a similar role in programming optic lobe NBs (Li et al., 2013) and exhibits dynamic expression in central brain NBs (data not shown), although its function there is not known. The fact that such genes have increased expression in Notch-induced NB tumours suggests a disruption to the temporal programme such that early expressed genes are transcribed at later times.

To substantiate the expression array results we compared the expression of *svp*, *cas* and *hth* in WT CNS with that in which NΔecd was induced for 24 h. No expression of Cas or Svp was detected in NBs in late WT CNS (Fig. 2F, Fig. S3A); their expression was entirely complementary to that of Dpn, suggesting that they were predominantly neuronal. Expression of Hth was more complex, with variable levels in NBs as well as in GMCs and in neuronal progeny (Fig. 2F). In the presence of NΔecd, all three proteins were detected in the ectopic NB-like cells. Many Dpn^+^ cells expressed Svp and Cas and they exhibited greatly enhanced Hth levels (Fig. 2F, Fig. S3A,B). Thus, unexpectedly, sustained Notch activity results in ectopic expression of temporal genes in late stage NBs.

By contrast, reductions in Notch function had relatively little impact on *cas*, *hth* or *svp* expression (Fig. 2G). In Type I lineages mutant for *Dl Ser*, Cas and Svp were still present, both at L3 in neurons (Fig. 2G, Table S4) and at earlier stages when these proteins are normally expressed in NBs (L2, 30-50 h after larval hatching; Fig. S3C). In *Dl Ser* mutant Type II lineages Hth and Svp were absent, suggesting that their expression might be dependent on Notch in these NBs. Thus, similar to the NB-specific genes, temporal programming genes are responsive to Notch overactivation but are largely resistant to Notch downregulation, except in Type II NBs, suggesting that compensatory mechanisms might be involved in regulating their NB expression.

### Su(H)-bound regions identify NB enhancers

**Fig. 3.**
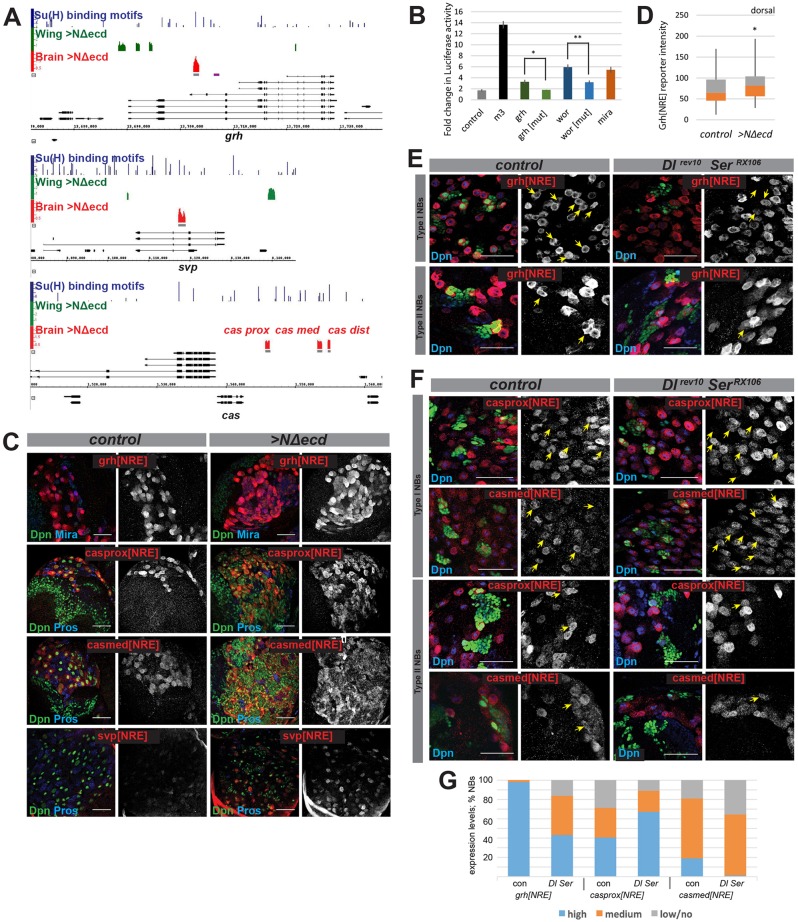
**Su(H)-bound regions identify NB enhancers.** (A) Genomic region spanning *grh*, *svp* and *cas* with graphs depicting Su(H)-bound regions (enrichment relative to input, AvgM, scale log_2_ 0-4) in NΔecd brains (red) and in NΔecd wing discs (green). Grey bars indicate regions tested for enhancer activity, and the purple bar indicates a previously identified *grh* NB enhancer. Details as in Fig. 1F. (B) Su(H)-bound regions from *grh*, *wor* and *mira* respond to Nicd in transient transfection assays. Fold change in expression from WT enhancers was significantly different from those with Su(H) binding motifs mutated. *P≤0.05, **P≤0.001, *t*-test. Average of three biological replicates; error bars indicate s.e.m. (C) Su(H)-bound region from *grh*, *cas* and *svp* direct NB-specific expression and respond to ectopic Notch activity. Expression from the indicated enhancers (red, white) in WT and NΔecd-expressing brain regions. Pros (blue) marks GMCs and neurons, and Mira (blue) marks NBs. (D) Intensity of expression from *grh[NRE]* in control and NΔecd-expressing dorsal brain NBs (20 NBs per brain lobe, five brains) was significantly different (*P<0.05, *t*-test). Box represents the interquartile range (IQR), orange/grey interface indicates median and whiskers indicate ±1.5×IQR. (E) *grh[NRE]* is sensitive to reduced Notch signalling. *grh[NRE]-lacZ* (red, white) in WT (green, left) or *Dl^rev10^ Ser^RX106^* (green, right). Yellow arrows indicate NBs from GFP-marked WT or mutant Type I and Type II lineages. (F) Enhancers from *cas* show differing sensitivity to reduced Notch signalling. Expression from the indicated enhancers (red, white) in WT (green, left) or *Dl^rev10^ Ser^RX106^* (green, right) clones. Yellow arrows indicate NBs from clonally marked WT or mutant Type I and Type II lineages. (G) Percentages of Type I NBs with high (blue), medium (orange) or low/no (grey) levels of expression of the indicated reporters in control versus *Dl^rev10^ Ser^RX106^* lineages. Scale bars: 50 μm.

If Notch coordinates the expression of stem cell and temporal programming genes, the regions occupied by Su(H) should correspond to Notch-regulated enhancers that direct expression in NBs. To test this, we first measured the activity of fragments encompassing the Su(H)-bound regions in *grh*, *wor* and *mira* using luciferase reporter assays (Fig. 3A,B). All three enhancers were upregulated by Nicd, and the responses of *grh* and *wor* were compromised when sequences corresponding to Su(H) recognition motifs were mutated, consistent with these being direct Notch-regulated enhancers (Fig. 3B). Subsequently, we focused on *grh*, where Su(H) binding was detected in an intronic region that was adjacent to, but not overlapping, a previously identified NB enhancer (Prokop et al., 1998). When placed upstream of a *lacZ* reporter, this fragment (*grh[NRE]*; Fig. 3A) was sufficient to direct expression in NBs (Fig. 3C,E). We therefore tested whether the enhancer was responsive to Notch signalling. First, expression of *grh[NRE]-lacZ* was detected in the ectopic NB-like cells in the presence of NΔecd (Fig. 3C). Its expression was also elevated within NΔecd NB-like cells as compared with neighbouring WT NBs in the dorsal brain (Fig. 3D). Second, *grh[NRE]-lacZ* expression was significantly reduced in NB lineages in which Notch signalling was compromised by mutations in *Dl* and *Ser* (Fig. 3E,G). Thus, the Su(H)-bound region within *grh* corresponds to an enhancer that specifically directs expression in NBs and that is responsive to Notch signalling, supporting the proposal that *grh* has a direct input from Notch activity.

A similar strategy was taken with the temporal factors *svp* and *cas*. Of three fragments bound by Su(H) in the vicinity of *cas* (Fig. 3A), two directed expression in NBs. *cas-prox[NRE]* generated high levels of *lacZ* expression in NBs, even at late larval stages, whereas *cas-med[NRE]* yielded lower levels of expression (Fig. 3C). The third region overlaps a previously defined NB enhancer (Kuzin et al., 2012), although it was inactive in our assays. Similarly, the *svp[NRE]* fragment (Fig. 3A) generated high levels of *lacZ* expression in a subset of NBs and lower levels in others (Fig. 3C). Thus, as with *grh*, the Su(H)-bound regions correspond to NB enhancers. Notably, however, these were still active in late NBs, a developmental stage when the corresponding genes would be shut off. This suggests either that these enhancers lack sequences necessary for their inactivation at later stages or that there is an enhancer handover mechanism (Boukhatmi et al., 2012), with a distinct region involved in mediating the repression at late stages.

The *svp* and *cas* enhancers all responded positively to ectopic Notch activity. Thus, the ectopic NB-like cells exhibited robust expression from *cas-prox[NRE]*, *cas-med[NRE]* and *svp[NRE]* in the presence of NΔecd (Fig. 3C). Conversely, many NBs had reduced levels of *cas-med[NRE]* and *svp[NRE]* when Notch activity was compromised, although the effects on *cas-med[NRE]* were subtle (Fig. 3F,G). By contrast, a high proportion of NBs retained high levels of *cas-prox[NRE]* expression in *Dl Ser* mutant lineages. Nevertheless, both *cas* and *svp* are associated with at least one enhancer that exhibits a consistent response to Notch activity, in agreement with these genes having direct regulatory input. In addition, Su(H)-bound regions for eight other genes were similarly tested *in vivo*, of which seven yielded NB expression (data not shown).

### Role of stem cell identity genes in Notch-induced NB hyperplasia

**Fig. 4.**
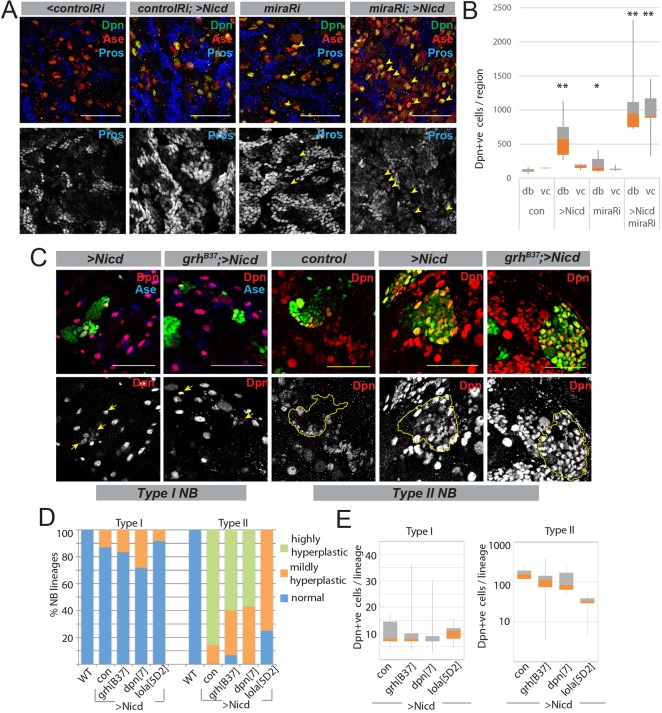
**Moderate consequences of removal of NB target genes on Notch-induced overproliferation.** (A) Depletion of Mira leads to Pros accumulation in NBs. Distribution of Pros (blue, white) in VNC NB lineages expressing control RNAi (controlRi) or *mira* RNAi (miraRi) with and without Nicd. Arrowheads mark NBs where Pros is detected in the nucleus. (B) Depletion of Mira exacerbates overproliferation. Number of Dpn^+^ cells in dorsal brains (db) and VNC (vc) of the genotypes indicated. Box represents IQR, orange/grey interface indicates median and whiskers indicate ±1.5×IQR. *P<0.05, **P<0.001 versus equivalent control (*t*-test). (C) Hyperplasia induced by Nicd in lineages lacking *grh* function. MARCM clones (green) overexpressing Nicd in WT and in *grh[B37]*, showing examples from Type I and Type II lineages. (Bottom row) Single channel showing Dpn^+^ cells; yellow arrows indicate NBs from marked clones, and yellow lines outline labelled lineages in some panels. (D) Percentage of Type I and Type II lineages exhibiting hyperplasia in the indicated genotypes. Hyperplasia is defined as >2 Dpn^+^ cells in Type I lineages and >28 Dpn^+^ cells in Type II lineages. (E) Number of Dpn^+^ cells present in overproliferating lineages (scored as in D) of the genotypes indicated. Box represents the IQR, orange/grey interface indicates median and whiskers indicate ±1.5×IQR. The reduced proliferation was only significant for lola[5D2]. Scale bars: 50 μm.

Possible roles for Notch-regulated NB-expressed genes might be to inhibit pro-differentiation factors and/or to maintain the self-renewal characteristics of NBs. One candidate for the former is *mira*, which encodes a protein required to retain key factors at the cytoplasmic cortex of NBs. In Type I NBs these include Pros, a transcriptional regulator of proliferation/differentiation, which can drive proliferating larval NBs into quiescence (Lai and Doe, 2014). By upregulating the expression of *mira*, Notch activity could favour the sequestration of Pros and so promote self-renewal. To test whether Mira is important for Notch-induced hyperplasia, we analysed the consequences of Mira depletion (by RNAi) in the background of Nicd overexpression (via *inscGal4;UAS-Nicd*, a combination that induces weaker hyperplasia than the NΔecd used in earlier experiments; Fig. 4A). Under these conditions, Pros was detectable in the nuclei of NBs, consistent with perturbation of Mira function (Fig. 4A). However, there was no reduction in hyperplasia; instead, the extent of hyperplasia was exacerbated (Fig. 4A,B, Fig. S4). Thus, the hyperplasia cannot be explained by Mira-mediated sequestration of factors such as Pros.

**Fig. 5.**
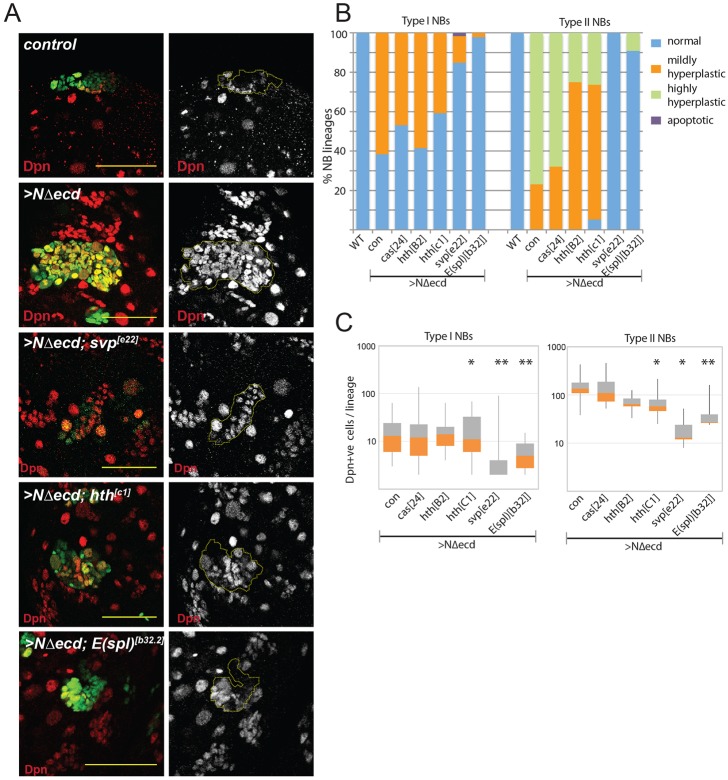
**Removal of temporal genes suppresses Notch-induced overproliferation.** (A) Hyperproliferation induced by NΔecd is suppressed in lineages lacking *svp* or *hth* function. Effects of *hth^C1^*, *svp*^e22^ and *E(spl)^b32.2^* on WT or NΔecd-expressing NB clones (green, MARCM; yellow lines in single-channel images outline marked lineages); examples are from Type II lineages. (B) Proportion of Type I and Type II lineages exhibiting hyperplasia in the indicated genotypes. (C) Number of Dpn^+^ cells in overproliferating lineages of the genotypes indicated. Box represents IQR, orange/grey interface indicates median and whiskers indicate ±1.5×IQR. *P<0.01, **P<0.001 versus NΔecd alone (Wilcoxon rank-sum test). Scale bars: 50 μm.

We then tested whether hyperplasia is reduced when the NB-specific TFs are depleted, since previous studies showed that deletion of *E(spl)m8-HLH* from *NΔecd*-overexpressing clones drastically reduces overproliferation (Zacharioudaki et al., 2012) (see Fig. 5A). However, little rescue of the overproliferation occurred in Type I lineages when *grh* or *dpn* were mutant (Fig. 4C-E, Table S5), nor when *wor* was depleted (Fig. S5B). The number and extent of overproliferating Nicd-expressing lineages that were mutant for *dpn* (28.2% of lineages, median of 2 Dpn^+^ cells, *n*=234) or *grh* (16.6% of lineages, median of 3 Dpn^+^ cells, *n*=229) was similar to Nicd alone (13% of lineages, median of 3 Dpn^+^ cells, *n*=355).

We also tested the effects of eliminating the widely expressed TF Lola, since several of its isoforms were upregulated by NΔecd. A strong hypomorphic mutation affecting all *lola* isoforms (*lola^5D2^*) had little impact on Type I lineage overproliferation (8.4% of lineages overproliferating, median of 6 Dpn^+^ cells, *n*=143). By contrast, the phenotypes in Type II lineages were partially suppressed by mutations affecting these genes (Fig. 4C-E, Table S5). For example, loss of *grh* decreased the Nicd-induced overproliferation phenotype from 158 Dpn^+^ cells per Type II lineage (*n*=21) to 114 Dpn^+^ cells per lineage (*n*=15), and *lola^5D2^* significantly rescued the Nicd phenotype (33 Dpn^+^ cells, *n*=8; WT lineages have 27 Dpn^+^ cells). A *dpn* allele also decreased overproliferation of Type II NBs, as observed previously (Zacharioudaki et al., 2012), yielding a median of 84 Dpn^+^ cells (*n*=7), but as there was considerable variability the effect was not statistically significant in these experiments.

Mutations in the NB-specific genes might not alter Notch-induced hyperplasia if they have overlapping functions in stem cell renewal, as previously shown for *dpn* and *E(spl)-C* (Zacharioudaki et al., 2012). We therefore examined whether removal of ‘stemness’ regulators, alone or in pairs, results in any defects in a normal background. We generated NB lineages that were double mutant for different combinations of TFs [*grh E(spl)-C*, *wor E(spl)-C*, *grh dpn*, *wor dpn* and *grh wor*] and measured changes in the number of progeny for both Type I and Type II NBs. Of those tested, only *grh wor* double-mutant lineages showed significant differences in the number of INPs and GMCs compared with either single mutant alone (Fig. S5). In addition, the *grh wor* Type I NBs had smaller nuclei (Fig. S5). Thus, these two NB-specific TFs have overlapping roles in maintaining NB size and proliferative rate. This suggests a robustness in the transcriptional network regulating stem cell characteristics, which might explain why removal of a single factor is insufficient to fully suppress Notch-induced hyperplasia.

### Role of temporal genes in Notch-induced hyperplasia

To decipher whether persistent expression of the temporal programming factors *svp*, *cas* and *hth* is important for NB hyperplasia, we used mutations to compromise their function while at the same time expressing NΔecd. Results showed that the hyperplasia in Type II lineages was highly susceptible to depletion of these TFs. Thus, the NΔecd-induced hyperplasia in Type II lineages (median of 135 Dpn^+^ cells/lineage, *n*=13) was rescued by removing either of the temporal genes *svp* or *hth* as well as by removing *E(spl)-C* (Fig. 5A-C, Table S5). The effects of removing *svp* were most dramatic: none of the lineages showed residual overproliferation and most lineages even had fewer Dpn^+^ cells (median 13 Dpn^+^ cells, *n*=8; Fig. 5C) than WT (median 28 Dpn^+^ cells). One way to reconcile this highly penetrant phenotype with the observation that only a subset of NB-like cells exhibit Svp expression is that the immunofluorescence only gives a snapshot of the expression at any given moment; many more NB-like cells might switch *svp* on at some stage to prompt the overproliferation. Alternatively, as we were only able to recover a relatively small number of clones of this genotype, it is possible that the phenotypic effects are overestimated. Eliminating *hth* in lineages expressing NΔecd also ameliorated the hyperplasia (Fig. 5B,C), with the median number of Dpn^+^ cells reduced by both *hth^B2^* (64.5 Dpn^+^ cells, *n*=8) and *hth^C1^* (58 Dpn^+^ cells, *n*=19). Finally, mutations affecting *cas* also resulted in decreased numbers of Dpn^+^ cells per Type II lineage (89 Dpn^+^ cells, *n*=25; Fig. 5C) although, because of the high variability, the effects were not statistically significant.

The effects on Type I lineages were less pronounced (Fig. 5B,C, Fig. S6). In these lineages, which were scored as hyperplastic when they contained two or more Dpn^+^ cells, the overexpression of *NΔecd* caused hyperplasia in 61.6% of lineages (*n*=86), with a median of 13 Dpn^+^ NB-like cells (which were usually intermediate in size between a normal NB and a GMC). Of the genes tested, only mutations in *svp* significantly rescued the Type I hyperplasia, although to a smaller extent than removing *E(spl)-C* (Zacharioudaki et al., 2012) (Fig. 5B,C). Specifically, only 13.5% of *svp* lineages (*n*=654) remained overgrown, with a median of 2 Dpn^+^ cells, although there was considerable variability. In addition, in a few lineages (1.7%) the NBs appeared to have undergone apoptosis. By contrast, mutations in *cas* or *hth* failed to significantly alter Type I NB hyperplasia; 47% *cas* (*n*=228), 40.8% *hth^C1^* (*n*=94) and 58.5% *hth^B12^* (*n*=174) lineages exhibited hyperplasia, with medians of 12, 14 and 11 Dpn^+^ cells, respectively.

### Expression of *wor* and of temporal genes is sufficient to induce mild hyperplasia

**Fig. 6.**
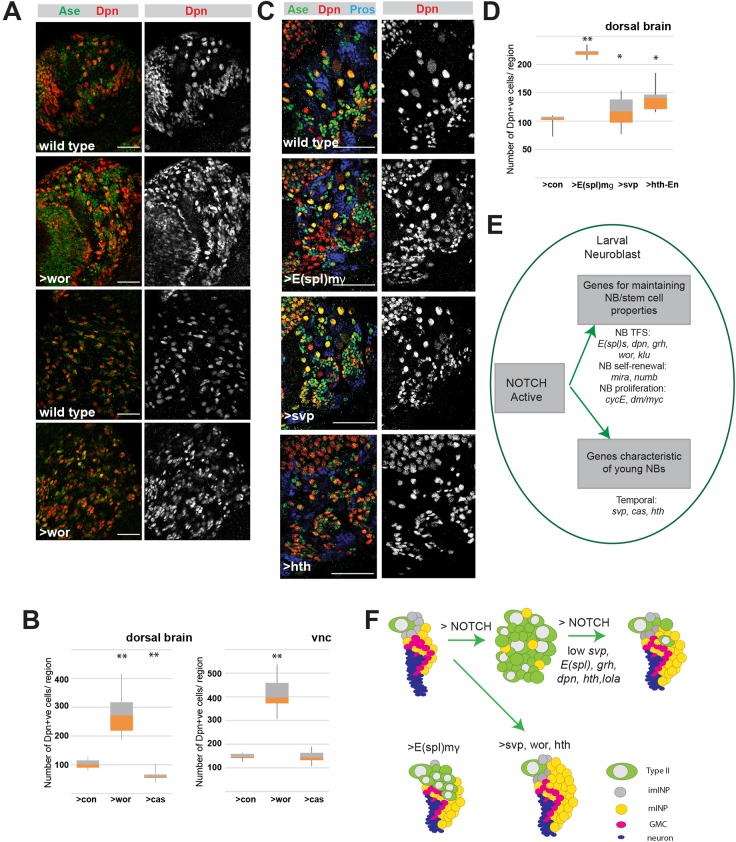
**Expression of *wor* or of temporal genes is sufficient to induce mild hyperplasia.** (A) *wor* expression (via *inscGal4*) induces hyperplasia in all regions (dorsal brain, top two rows; VNC, bottom two rows). Ase (green), Dpn (red or white). (B) Phenotypes from expression of *wor* or *cas* compared with control (*UAS-lacZ*). Total numbers of Dpn^+^ cells were scored in dorsal brain and VNC. **P<0.001 versus equivalent control (*t*-test). Similar results were obtained in ventral brains (data not shown). (C) Dorsal brain from the indicated genotypes, where transgenes were ectopically expressed via *inscGal4*. Anterior is inferior, lateral is leftwards; Ase, green; Dpn, red or white; Pros, blue. E(spl)mγ-HLH leads to a large increase in Dpn^+^ Ase^−^ cells; Svp or En-Hth leads to an increase in the number of Dpn^+^ Ase^+^ cells. (D) Number of Dpn^+^ cells in dorsal brains overexpressing the proteins indicated, with β-galactosidase as control (con). *P<0.05, **P<0.001 versus equivalent control (*t*-test). (E) Summary of the Notch response in NBs. (F) Diagram illustrating the phenotypes (imINP, immature INP; mINP, mature INP) in Type II NB lineages caused by Notch and how these are influenced by manipulating the identified Notch targets (summarised in Table S5). Scale bars: 50 μm.

From the loss-of-function experiments it appears that several Notch-regulated genes contribute to the hyperplasia. To investigate whether any of these targets is sufficient to induce excess NBs, we assessed the consequences of their overexpression in larval lineages by scoring the numbers of Dpn^+^ cells (Fig. 6). We note that in some cases [*grh*, *E(spl)mγ-HLH*, *wor*] these manipulations would augment existing expression levels, whereas for *svp* they would result in ectopic expression.

Two of the three NB-specific genes tested resulted in significant hyperplasia. Notably, expression of *wor* was sufficient to promote an increase in NB-like cells both in the dorso-posterior brain, where Type II lineages reside, and in the ventral nerve cord (VNC), where Type I lineages occur (Fig. 6A,B). As previously shown, overexpression of *E(spl)mγ-HLH* also caused hyperplasia, primarily in the dorso-posterior brain, where it generated regions of contiguous Dpn^+^ Ase^−^ cells. Their size was intermediate between an INP and an NB (Fig. 6C) and they most likely arose from Type II lineages. By contrast, *grh* overexpression failed to cause any increase in NB numbers, even in these more susceptible Type II lineages (data not shown).

Whereas expression of Cas had no effect on NB lineages (Fig. 6B, Fig. S7A), overexpression of Svp did elicit supernumerary Dpn^+^ cells in Type II lineages (Fig. 6C,D, Fig. S7B). These were Ase^+^ and generally smaller than NBs (Fig. 6C). Excess Dpn^+^ cells were also produced following expression of a chimeric Hth, in which Hth is fused to the Engrailed (En) repressor domain to create a constitutive Hth repressor. These excess Dpn^+^ cells were Ase^+^ and were similar to, or smaller than, mature INPs. In addition, some hyperplasia was present in Type I NB lineages (Fig. 6C,D). As Hth has been reported to have both activator and repressor functions (Inbal et al., 2001; Wernet and Desplan, 2014), we cannot predict whether En-Hth would act as a positive or a dominant-negative factor in the conditions tested. Regardless of the mechanisms involved, altered activity of Svp and Hth is nevertheless sufficient to induce a modest level of hyperplasia, especially in Type II lineages.

## DISCUSSION

Notch activity is sustained in post-embryonic NBs throughout their lifetime and, when activated inappropriately, is sufficient to confer NB-like properties on the progeny. Our analysis of the genes regulated by Notch under these circumstances reveals that it is likely to achieve its functions through multi-pronged regulation of the stem cell programme. Of the 246 putative direct Notch target genes that we identified in hyperplastic CNS, the majority (>55%) are enriched in the NB transcriptome and 28% are assigned to the NB network regulated by Ase. Many of these encode TFs that have been implicated in stem cell regulation, such as *grh*, *klu*, *wor*, *dpn* and *E(spl)mγ-HLH*, and others encode proteins involved in regulating asymmetric stem cell divisions, including *mira* and *numb*. An unexpected constituent of the Notch-upregulated genes were those implicated in the temporal programming of NBs, represented by *svp*, *cas* and *hth*. Such genes are thought to confer stage-specific NB characteristics and determine the ultimate timing of their cell cycle exit. For example, in larval NBs, *svp* and *cas* are expressed early in L2 and, if absent, a switch in neuronal identities fails to occur and the NBs fail to cease proliferating appropriately (Maurange et al., 2008). Similarly, *hth* has been implicated in the temporal cascade of optic lobe larval NBs (Li et al., 2013). By promoting the re-expression or extended expression of factors that are normally present transiently, Notch activity is likely to change the NB developmental clock. Sustained expression of such factors may perturb the ability of NBs to exit the cell cycle correctly. This would imply that persistent expression of such factors has similarly disruptive consequences to their total removal (Maurange et al., 2008). As Notch-regulated enhancers were enriched in target motifs for both E(spl)mγ-HLH and Hth, feed-forward regulatory loops might also be important in the regulation of NB maintenance and progression by Notch, although the mechanisms remain to be deciphered.

With the exception of *mira*, all of the Notch targets tested were found to contribute to Notch-induced hyperplasia. Type II NB hyperplasia was more sensitive to the attenuation of target genes, with all mutations reducing the overgrowth to a greater or lesser extent, whereas Type I NB hyperplasia was only ameliorated by *E(spl)-C* and *svp*. Furthermore, a subset, notably *wor*, *svp*, *hth*, *dpn*, *E(spl)mγ-HLH* and *klu*, were themselves sufficient to drive mild hyperplasia, especially in Type II lineages (see also Berger et al., 2012; San-Juán and Baonza, 2011; Xiao et al., 2012; Zacharioudaki et al., 2012). Thus, both stemness and temporal TFs cooperate to sustain Notch-induced hyperplasia. Furthermore, although individual factors each make some unique contribution to NB maintenance, several [E(spl)mγ-HLH and Dpn, Grh and Wor] appear to have overlapping functions, which confers robustness on the regulatory network.

The regions bound by Su(H) identified NB enhancers, in agreement with the nearby genes being targets of Notch in NBs. However, an unexpected feature was that the NB expression of these genes did not appear to be strictly dependent on Notch activity. This might in part be explained by the regulated genes having multiple NB enhancers; for example, at least two further NB-specific enhancers have been identified for *grh*, besides *grh[NRE]* (Brody et al., 2012; Prokop et al., 1998). However, this cannot fully account for the lack of Notch dependency. Even when we focused on individual enhancers, loss of Notch signalling did not always eliminate their expression, although in most cases it did reduce it, as observed previously for *E(spl)m8-HLH* (Zacharioudaki et al., 2012). The only target gene tested that is fully dependent on Notch for expression in NBs, being undetectable upon Notch loss of function, is *E(spl)mγ-HLH* (Almeida and Bray, 2005; Zacharioudaki et al., 2012). It thus appears that in NBs many of the Notch targets respond to additional transcriptional cues that can partially compensate for the absence of Notch. It will be interesting to determine whether this is also true for the majority of Notch target enhancers in other tissues.

Persistent Notch activity in several other tissues also causes extreme hyperplasia. Surprisingly, however, only 18 of the genes upregulated in the NB hyperplasia are also targeted in an epithelial hyperplasia caused by ectopic activity of Notch in wing discs. These include HES/*E(spl)* genes, which respond robustly to Notch signalling in most cellular contexts, and the growth regulator *Myc*, which is also a common target even in human pathologies, such as T-ALL (Klinakis et al., 2006; Weng et al., 2006). Thus, the mechanisms through which Notch induces hyperplasia differ in the two contexts. It is possible that this relates to the fact that the hyperplasia originates from a stem cell lineage in one case and an epithelium in the other. Indeed, comparisons between the genes regulated in the epithelial model and those regulated by Notch1 in a breast cancer cell line revealed a surprising extent of overlap (Djiane et al., 2013; Mazzone et al., 2010). It will therefore be important in future to make comparisons with different stem cell-related hyperplasias in mammals, to ask whether the Notch-regulated genes exhibit similar characteristics to those observed for NBs. Such comparisons will help to ascertain whether there are indeed common themes in the transcriptional outputs from Notch activation that reflect the different cellular contexts.

## MATERIALS AND METHODS

### *Drosophila* genetics

*Drosophila* stocks are described in FlyBase and were obtained from the Bloomington Stock Center unless otherwise indicated. Overproliferating third instar larval CNS was generated by crossing *tubGal80ts; UASNΔecd* flies with *UASCD8GFP; grhNB-Gal4* flies to drive expression in most NBs (Prokop et al., 1998). Crosses were kept at 18°C for 10 days, then transferred to 30°C for 24 h prior to dissection.

Stocks for generating Notch loss of function, loss-of-function mutants coupled with hyperactivation of Notch, and double-mutant combinations are detailed in the supplementary Materials and Methods and were crossed to appropriate *FRT aTub-Gal80* counter-chromosomes combined with *hs-FLP*, *aTub-Gal4*, *UAS-GFP* for generating clones. WT *FRT* chromosomes (e.g. *FRT82B πMyc* or *FRTG13*) were used for control clones. Progeny underwent heatshock for 1 h at 37°C at ∼72 h after egg lay (AEL). For experiments involving RNAi, progeny were transferred to 30°C for 72 h prior to dissection (to enhance RNAi activity). Phenotypes were analysed at late L3 (∼110-120 h AEL).

For overexpression of genes in NBs, the UAS-lines *UAS-lacZ* (control), *UAS-E(spl)mγ* (Ligoxygakis et al., 1998), *UAS-svp* (Kerber et al., 1998), *UAS-EN::Hth* (Inbal et al., 2001), *UAS-Wor* (Berger et al., 2012) were crossed to *inscGal4; tubGal80ts* and the progeny were transferred to 30°C for 72 h prior to dissection.

### Expression arrays and genome-wide ChIP

For each expression array, RNA was isolated from the CNS of 300 flies using the RNeasy Plus Mini Kit (Qiagen) and reverse transcribed using standard procedures before hybridising to Affymetrix GeneChip *Drosophila* Genome 2.0 Array 3 (see the supplementary Materials and Methods for details). Four replicate arrays were analysed for each genotype. Quantile normalised data were analysed using Limma (Smyth, 2004) to estimate the log_2_ FC in Notch versus control samples and the Benjamini-Hochberg adjusted *P*-values (FDR). Data from expression arrays have been deposited in ArrayExpress (E-MTAB-3561).

For each ChIP, chromatin was prepared from the CNS of 50 flies and the Su(H) ChIP performed as described previously (Krejcí et al., 2009). The products were amplified and hybridised to NimbleGen *D. melanogaster* 2.1 M Whole-Genome Tiling Arrays. Three biological replicates were performed and quantile normalisation was applied to replicates for each genotype. Bound regions (peaks) were identified using Tamalpais at T02P005 stringency [top 2%, *P*≤0.005 (Bieda et al., 2006)]. Genes in proximity to ChIP peaks were identified by the nearest genes upstream and downstream of each peak (using coordinates for the gene body) with a distance cutoff of 20 kb. ChIP data have been deposited in Gene Expression Omnibus (GEO series GSE68614).

Gene set enrichment analysis (Subramanian et al., 2005) was performed using the ‘pre-ranked’ option to analyse the gene list ranked by log_2_ FC from the mRNA expression data (3717 genes, FDR≤0.1) for enrichment of Su(H)-bound genes. As recommended, the more conservative ‘classic’ scoring approach was used, which computes enrichment using only the gene’s ranking (with no increment for the absolute value of the ranking metric).

GO analysis was performed using the DAVID bioinformatics resource (Huang et al., 2009). Motif enrichment analysis utilised the Bioconductor package PWMEnrich, which assesses the enrichment of each motif from a library of 650 experimentally derived *Drosophila* TF DNA motifs (Stojnic and Diez, 2014). Enrichments for NB-expressed genes, Ase targets, and NB stem cell networks were assessed using Fisher's exact test, with the Benjamini-Hochberg method (Benjamini and Hochberg, 1995) to correct for multiple sampling.

### NRE reporters

Putative Notch-regulated enhancers (NREs) in *grh*, *svp* and *cas* (coordinates are provided in the supplementary Materials and Methods) were cloned in pBlueRabbit (pBR) (Housden et al., 2012). Flies carrying the pBR transgenes were generated by ɸC31-mediated site-directed integration in the attP40 landing site.

### Immunofluorescence

Fixation and immunohistochemistry of larval tissues were performed according to standard protocols. Details of primary antibodies are provided in the supplementary Materials and Methods. Mouse, rabbit, guinea pig or rat secondary antibodies were conjugated to Alexa 488, 555, 568, 633 or 647 (Molecular Probes) or to FiTC, Cy3 or Cy5 (Jackson ImmunoResearch). Samples were imaged on a Leica SP2 or TCS SP8 confocal microscope (Confocal Facility, University of Crete or CAIC, University of Cambridge).

## References

[DEV126326C1] Almeida M. S. and Bray S. J. (2005). Regulation of post-embryonic neuroblasts by Drosophila Grainyhead. *Mech. Dev.* 122, 1282-1293. 10.1016/j.mod.2005.08.004 16275038

[DEV126326C2] Babaoglan A. B., O'Connor-Giles K. M., Mistry H., Schickedanz A., Wilson B. A. and Skeath J. B. (2009). Sanpodo: a context-dependent activator and inhibitor of Notch signaling during asymmetric divisions. *Development* 136, 4089-4098. 10.1242/dev.040386 19906847 PMC2781049

[DEV126326C3] Babaoğlan A. B., Housden B. E., Furriols M. and Bray S. J. (2013). Deadpan contributes to the robustness of the notch response. *PLoS ONE* 8, e75632 10.1371/journal.pone.0075632 24086596 PMC3782438

[DEV126326C4] Badenhorst P. (2001). Tramtrack controls glial number and identity in the Drosophila embryonic CNS. *Development* 128, 4093-4101. 11641231 10.1242/dev.128.20.4093

[DEV126326C5] Bayraktar O. A. and Doe C. Q. (2013). Combinatorial temporal patterning in progenitors expands neural diversity. *Nature* 498, 449-455. 10.1038/nature12266 23783519 PMC3941985

[DEV126326C6] Bello B. C., Izergina N., Caussinus E. and Reichert H. (2008). Amplification of neural stem cell proliferation by intermediate progenitor cells in Drosophila brain development. *Neural Dev.* 3, 5 10.1186/1749-8104-3-5 18284664 PMC2265709

[DEV126326C7] Benjamini Y. and Hochberg Y. (1995). Controlling the false discovery rate: a practical and powerful approach to multiple testing on JSTOR. *J. R. Stat. Soc. Ser. B* 57, 289-300.

[DEV126326C8] Berger C., Harzer H., Burkard T. R., Steinmann J., van der Horst S., Laurenson A.-S., Novatchkova M., Reichert H. and Knoblich J. A. (2012). FACS purification and transcriptome analysis of drosophila neural stem cells reveals a role for Klumpfuss in self-renewal. *Cell Rep.* 2, 407-418. 10.1016/j.celrep.2012.07.008 22884370 PMC3828055

[DEV126326C9] Bieda M., Xu X., Singer M. A., Green R. and Farnham P. J. (2006). Unbiased location analysis of E2F1-binding sites suggests a widespread role for E2F1 in the human genome. *Genome Res.* 16, 595-605. 10.1101/gr.4887606 16606705 PMC1457046

[DEV126326C10] Boone J. Q. and Doe C. Q. (2008). Identification of Drosophila type II neuroblast lineages containing transit amplifying ganglion mother cells. *Dev. Neurobiol.* 68, 1185-1195. 10.1002/dneu.20648 18548484 PMC2804867

[DEV126326C12] Boukhatmi H., Frendo J. L., Enriquez J., Crozatier M., Dubois L. and Vincent A. (2012). Tup/Islet1 integrates time and position to specify muscle identity in Drosophila. *Development* 139, 3572-3582. 10.1242/dev.083410 22949613

[DEV126326C13] Bowman S. K., Rolland V., Betschinger J., Kinsey K. A., Emery G. and Knoblich J. A. (2008). The tumor suppressors Brat and Numb regulate transit-amplifying neuroblast lineages in Drosophila. *Dev. Cell* 14, 535-546. 10.1016/j.devcel.2008.03.004 18342578 PMC2988195

[DEV126326C14] Bray S. J. (2006). Notch signalling: a simple pathway becomes complex. *Nat. Rev. Mol. Cell Biol.* 7, 678-689. 10.1038/nrm2009 16921404

[DEV126326C15] Brody T., Yavatkar A. S., Kuzin A., Kundu M., Tyson L. J., Ross J., Lin T.-Y., Lee C.-H., Awasaki T., Lee T. et al. (2012). Use of a Drosophila genome-wide conserved sequence database to identify functionally related cis-regulatory enhancers. *Dev. Dyn.* 241, 169-189. 10.1002/dvdy.22728 22174086 PMC3243966

[DEV126326C16] Capaccione K. M. and Pine S. R. (2013). The Notch signaling pathway as a mediator of tumor survival. *Carcinogenesis* 34, 1420-1430. 10.1093/carcin/bgt127 23585460 PMC3697894

[DEV126326C17] Cenci C. and Gould A. P. (2005). Drosophila Grainyhead specifies late programmes of neural proliferation by regulating the mitotic activity and Hox-dependent apoptosis of neuroblasts. *Development* 132, 3835-3845. 10.1242/dev.01932 16049114

[DEV126326C18] Chai P. C., Liu Z., Chia W. and Cai Y. (2013). Hedgehog signaling acts with the temporal cascade to promote neuroblast cell cycle exit. *PLoS Biol.* 11, e1001494 10.1371/journal.pbio.1001494 23468593 PMC3582610

[DEV126326C19] Connor-giles K. M. O., Skeath J. B. and Louis S. (2003). Numb inhibits membrane localization of sanpodo, a four-pass transmembrane protein, to promote asymmetric divisions in Drosophila. *Dev. Cell* 5, 231-243. 12919675 10.1016/s1534-5807(03)00226-0

[DEV126326C20] Djiane A., Krejci A., Bernard F., Fexova S., Millen K. and Bray S. J. (2013). Dissecting the mechanisms of Notch induced hyperplasia. *EMBO J.* 32, 60-71. 10.1038/emboj.2012.326 23232763 PMC3545308

[DEV126326C21] Guo M., Jan L. Y. and Jan Y. N. (1996). Control of daughter cell fates during asymmetric division: interaction of Numb and Notch. *Neuron* 17, 27-41. 10.1016/S0896-6273(00)80278-0 8755476

[DEV126326C22] Hertz G. Z. and Stormo G. D. (1999). Identifying DNA and protein patterns with statistically significant alignments of multiple sequences. *Bioinformatics* 15, 563-577. 10.1093/bioinformatics/15.7.563 10487864

[DEV126326C24] Homem C. C. F., Steinmann V., Burkard T. R., Jais A., Esterbauer H. and Knoblich J. A. (2014). Ecdysone and mediator change energy metabolism to terminate proliferation in Drosophila neural stem cells. *Cell* 158, 874-888. 10.1016/j.cell.2014.06.024 25126791

[DEV126326C25] Housden B. E., Millen K. and Bray S. J. (2012). Drosophila reporter vectors compatible with ΦC31 integrase transgenesis techniques and their use to generate new notch reporter fly lines. *G3* 2, 79-82. 10.1534/g3.111.001321 22384384 PMC3276196

[DEV126326C26] Huang D. W., Sherman B. T. and Lempicki R. A. (2009). Systematic and integrative analysis of large gene lists using DAVID bioinformatics resources. *Nat. Protoc.* 4, 44-57. 10.1038/nprot.2008.211 19131956

[DEV126326C27] Inbal A., Halachmi N., Dibner C., Frank D. and Salzberg A. (2001). Genetic evidence for the transcriptional-activating function of Homothorax during adult fly development. *Development* 128, 3405-3413. 11566847 10.1242/dev.128.18.3405

[DEV126326C28] Izergina N., Balmer J., Bello B. and Reichert H. (2009). Postembryonic development of transit amplifying neuroblast lineages in the Drosophila brain. *Neural Dev.* 4, 44 10.1186/1749-8104-4-44 20003348 PMC2801669

[DEV126326C30] Kang K. H. and Reichert H. (2014). Control of neural stem cell self-renewal and differentiation in Drosophila. *Cell Tissue Res.* 359, 33-45. 10.1007/s00441-014-1914-9 24902665

[DEV126326C31] Kerber B., Fellert S. and Hoch M. (1998). Seven-up, the Drosophila homolog of the COUP-TF orphan receptors, controls cell proliferation in the insect kidney. *Genes Dev.* 12, 1781-1786. 10.1101/gad.12.12.1781 9637680 PMC316909

[DEV126326C32] Klinakis A., Szabolcs M., Politi K., Kiaris H., Artavanis-Tsakonas S. and Efstratiadis A. (2006). Myc is a Notch1 transcriptional target and a requisite for Notch1-induced mammary tumorigenesis in mice. *Proc. Natl. Acad. Sci. USA* 103, 9262-9267. 10.1073/pnas.0603371103 16751266 PMC1570422

[DEV126326C33] Knoblich J. A. (2008). Mechanisms of asymmetric stem cell division. *Cell* 132, 583-597. 10.1016/j.cell.2008.02.007 18295577

[DEV126326C34] Kopan R. and Ilagan M. X. G. (2009). The canonical Notch signaling pathway: unfolding the activation mechanism. *Cell* 137, 216-233. 10.1016/j.cell.2009.03.045 19379690 PMC2827930

[DEV126326C35] Krejcí A., Bernard F., Housden B. E., Collins S. and Bray S. J. (2009). Direct response to Notch activation: signaling crosstalk and incoherent logic. *Sci. Signal.* 2, ra1 10.1126/scisignal.2000140 19176515

[DEV126326C36] Kuzin A., Kundu M., Ross J., Koizumi K., Brody T. and Odenwald W. F. (2012). The cis-regulatory dynamics of the Drosophila CNS determinant castor are controlled by multiple sub-pattern enhancers. *Gene Expr. Patterns* 12, 261-272. 10.1016/j.gep.2012.05.004 22691242 PMC3436978

[DEV126326C37] Lai S.-L. and Doe C. Q. (2014). Transient nuclear Prospero induces neural progenitor quiescence. *Elife* 3, e03363 10.7554/elife.03363 PMC4212206 25354199

[DEV126326C38] Le Borgne R., Bardin A. and Schweisguth F. (2005). The roles of receptor and ligand endocytosis in regulating Notch signaling. *Development* 132, 1751-1762. 10.1242/dev.01789 15790962

[DEV126326C39] Li X., Erclik T., Bertet C., Chen Z., Voutev R., Venkatesh S., Morante J., Celik A. and Desplan C. (2013). Temporal patterning of Drosophila medulla neuroblasts controls neural fates. *Nature* 498, 456-462. 10.1038/nature12319 23783517 PMC3701960

[DEV126326C40] Ligoxygakis P., Yu S. Y., Delidakis C. and Baker N. E. (1998). A subset of notch functions during Drosophila eye development require Su(H) and the E(spl) gene complex. *Development* 125, 2893-2900. 9655811 10.1242/dev.125.15.2893

[DEV126326C41] Maurange C., Cheng L. and Gould A. P. (2008). Temporal transcription factors and their targets schedule the end of neural proliferation in Drosophila. *Cell* 133, 891-902. 10.1016/j.cell.2008.03.034 18510932

[DEV126326C42] Mazzone M., Selfors L. M., Albeck J., Overholtzer M., Sale S., Carroll D. L., Pandya D., Lu Y., Mills G. B., Aster J. C. et al. (2010). Dose-dependent induction of distinct phenotypic responses to Notch pathway activation in mammary epithelial cells. *Proc. Natl. Acad. Sci. USA* 107, 5012-5017. 10.1073/pnas.1000896107 20194747 PMC2841923

[DEV126326C43] Mootha V. K., Lindgren C. M., Eriksson K.-F., Subramanian A., Sihag S., Lehar J., Puigserver P., Carlsson E., Ridderstråle M., Laurila E. et al. (2003). PGC-1alpha-responsive genes involved in oxidative phosphorylation are coordinately downregulated in human diabetes. *Nat. Genet.* 34, 267-273. 10.1038/ng1180 12808457

[DEV126326C44] Ntziachristos P., Lim J. S., Sage J. and Aifantis I. (2014). From fly wings to targeted cancer therapies: a centennial for notch signaling. *Cancer Cell* 25, 318-334. 10.1016/j.ccr.2014.02.018 24651013 PMC4040351

[DEV126326C45] Prokop A., Bray S., Harrison E. and Technau G. M. (1998). Homeotic regulation of segment-specific differences in neuroblast numbers and proliferation in the Drosophila central nervous system. *Mech. Dev.* 74, 99-110. 10.1016/S0925-4773(98)00068-9 9651493

[DEV126326C46] Reeves N. and Posakony J. W. (2005). Genetic programs activated by proneural proteins in the developing Drosophila PNS. *Dev. Cell* 8, 413-425. 10.1016/j.devcel.2005.01.020 15737936

[DEV126326C47] Rhyu M. S., Jan L. Y. and Jan Y. N. (1994). Asymmetric distribution of numb protein during division of the sensory organ precursor cell confers distinct fates to daughter cells. *Cell* 76, 477-491. 10.1016/0092-8674(94)90112-0 8313469

[DEV126326C48] San-Juán B. P. and Baonza A. (2011). The bHLH factor deadpan is a direct target of Notch signaling and regulates neuroblast self-renewal in Drosophila. *Dev. Biol.* 352, 70-82. 10.1016/j.ydbio.2011.01.019 21262215

[DEV126326C49] Skalska L., Stojnic R., Li J., Fischer B., Cerda-Moya G., Sakai H., Tajbakhsh S., Russell S., Adryan B. and Bray S. J. (2015). Chromatin signatures at Notch-regulated enhancers reveal large-scale changes in H3K56ac upon activation. *EMBO J.* 34, 1889-1904. 10.15252/embj.201489923 26069324 PMC4547894

[DEV126326C50] Smyth G. K. (2004). Linear models and empirical bayes methods for assessing differential expression in microarray experiments. *Stat. Appl. Genet. Mol. Biol.* 3, 1-25. 10.2202/1544-6115.1027 16646809

[DEV126326C51] Song Y. and Lu B. (2011). Regulation of cell growth by Notch signaling and its differential requirement in normal vs. tumor-forming stem cells in Drosophila. *Genes Dev.* 25, 2644-2658. 10.1101/gad.171959.111 22190460 PMC3248685

[DEV126326C52] Sousa-Nunes R. and Somers W. G. (2013). Mechanisms of asymmetric progenitor divisions in the Drosophila central nervous system. *Adv. Exp. Med. Biol.* 786, 79-102. 10.1007/978-94-007-6621-1_6 23696353

[DEV126326C53] Southall T. D. and Brand A. H. (2009). Neural stem cell transcriptional networks highlight genes essential for nervous system development. *EMBO J.* 28, 3799-3807. 10.1038/emboj.2009.309 19851284 PMC2770102

[DEV126326C54] Southall T. D., Gold K. S., Egger B., Davidson C. M., Caygill E. E., Marshall O. J. and Brand A. H. (2013). Cell-type-specific profiling of gene expression and chromatin binding without cell isolation: assaying RNA Pol II occupancy in neural stem cells. *Dev. Cell* 26, 101-112. 10.1016/j.devcel.2013.05.020 23792147 PMC3714590

[DEV126326C55] Spana E. P. and Doe C. Q. (1996). Numb antagonizes Notch signaling to specify sibling neuron cell fates. *Neuron* 17, 21-26. 10.1016/S0896-6273(00)80277-9 8755475

[DEV126326C56] Stojnic R. and Diez D. (2014). PWMEnrich: PWM enrichment analysis. R package version 4.2.0. http://bioconductor.jp/packages/3.0/bioc/html/PWMEnrich.html.

[DEV126326C57] Subramanian A., Tamayo P., Mootha V. K., Mukherjee S., Ebert B. L., Gillette M. A., Paulovich A., Pomeroy S. L., Golub T. R., Lander E. S. et al. (2005). Gene set enrichment analysis: a knowledge-based approach for interpreting genome-wide expression profiles. *Proc. Natl. Acad. Sci. USA* 102, 15545-15550. 10.1073/pnas.0506580102 16199517 PMC1239896

[DEV126326C58] Wang H., Somers G. W., Bashirullah A., Heberlein U., Yu F. and Chia W. (2006). Aurora-A acts as a tumor suppressor and regulates self-renewal of Drosophila neuroblasts. *Genes Dev.* 20, 3453-3463. 10.1101/gad.1487506 17182870 PMC1698451

[DEV126326C59] Weng A. P., Millholland J. M., Yashiro-Ohtani Y., Arcangeli M. L., Lau A., Wai C., del Bianco C., Rodriguez C. G., Sai H., Tobias J. et al. (2006). c-Myc is an important direct target of Notch1 in T-cell acute lymphoblastic leukemia/lymphoma. *Genes Dev.* 20, 2096-2109. 10.1101/gad.1450406 16847353 PMC1536060

[DEV126326C60] Weng M., Golden K. L. and Lee C.-Y. (2010). dFezf/Earmuff maintains the restricted developmental potential of intermediate neural progenitors in Drosophila. *Dev. Cell* 18, 126-135. 10.1016/j.devcel.2009.12.007 20152183 PMC6699514

[DEV126326C61] Wernet M. F. and Desplan C. (2014). Homothorax and Extradenticle alter the transcription factor network in Drosophila ommatidia at the dorsal rim of the retina. *Development* 141, 918-928. 10.1242/dev.103127 24496628 PMC3912834

[DEV126326C62] Xiao Q., Komori H. and Lee C.-Y. (2012). Klumpfuss distinguishes stem cells from progenitor cells during asymmetric neuroblast division. *Development* 139, 2670-2680. 10.1242/dev.081687 22745313 PMC3392700

[DEV126326C63] Zacharioudaki E., Magadi S. S. and Delidakis C. (2012). bHLH-O proteins are crucial for Drosophila neuroblast self-renewal and mediate Notch-induced overproliferation. *Development* 139, 1258-1269. 10.1242/dev.071779 22357926

[DEV126326C64] Zhu S., Wildonger J., Barshow S., Younger S., Huang Y. and Lee T. (2012). The bHLH repressor Deadpan regulates the self-renewal and specification of Drosophila larval neural stem cells independently of Notch. *PLoS ONE* 7, e46724 10.1371/journal.pone.0046724 23056424 PMC3466283

